# PI3K inhibition with alpelisib: a new hope for congenital hyperinsulinism

**DOI:** 10.1097/MS9.0000000000004604

**Published:** 2025-12-16

**Authors:** Mir Raza Ali, Abdul Khalique, Iman Osman Abufatima

**Affiliations:** a Department of Medicine, Jinnah Postgraduate Medical Centre, Karachi, Pakistan; b Faculty of Medicine, University of Medical Sciences and Technology, Khartoum, Sudan

*To the Editor,*

Congenital hyperinsulinism (CHI) remains one of the most challenging pediatric endocrine emergencies, marked by recurrent hypoglycemia due to inappropriate insulin secretion. Traditional therapies, including diazoxide, octreotide, and near-total pancreatectomy, often provide limited benefit and carry substantial morbidity^[1]^. Families and clinicians have long sought safer, more targeted alternatives.

The phosphoinositide 3-kinase (PI3K) pathway plays a central role in insulin signaling, and in specific CHI subtypes, pathway overactivation drives uncontrolled insulin release. Alpelisib’s inhibition of PI3K-alpha reduces downstream AKT activation, decreasing β-cell membrane depolarization and limiting glucose-stimulated insulin exocytosis, thereby stabilizing glucose homeostasis. Infants with severe, drug-resistant CHI may therefore benefit significantly from alpelisib, a selective PI3K-alpha inhibitor initially approved for oncology indications^[2]^.

Clinical reports describe rapid stabilization in children with life-threatening hypoglycemia who had not responded to octreotide or diazoxide^[2,3]^. This targeted modulation of insulin signaling rather than broad hormonal suppression represents an important mechanistic advancement in pediatric endocrinology. For families facing the prospect of surgical pancreatectomy, with lifelong risk of diabetes and exocrine insufficiency, such outcomes are profoundly meaningful^[3]^.

Preliminary observations suggest that alpelisib improves glucose stability and reduces hospitalizations, offering children a more predictable daily life^[4]^. These accounts highlight not only biochemical improvement but also the emotional impact of a therapy that transitions families from persistent fear to renewed hope.

As with any emerging therapy, long-term safety considerations remain. Monitoring is essential to understand effects on growth, immune function, and metabolic health^[4,5]^. Experience from oncology provides partial insight, but pediatric-specific caution is warranted. Early-phase interventional studies and ongoing international CHI registries are now collecting systematic data on dosing, durability of response, and safety profiles, strengthening the foundation for broader clinical use.

The use of alpelisib exemplifies the promise of mechanism-based precision medicine in pediatric endocrinology. It reflects a shift away from nonspecific suppression or irreversible surgery toward targeted correction of disease pathways. More than a pharmacologic innovation, it represents a meaningful step toward integrating molecular science with compassionate, individualized care for families living with CHI. TITAN guidelines: This manuscript complies with TITAN Guidelines, 2025, declaring no use of artificial intelligence^[6]^.

## Data Availability

Data sharing is not applicable to this article.

## Research registration unique identifying number (UIN)

Our study does not involve any human participants or human data; therefore, registration in a study registry is not applicable.
